# Effect of Zirconia Nanoparticles in Epoxy-Silica Hybrid Adhesives to Join Aluminum Substrates

**DOI:** 10.3390/ma10101135

**Published:** 2017-09-27

**Authors:** José de Jesús Figueroa-Lara, Miguel Torres-Rodríguez, Mirella Gutiérrez-Arzaluz, Mario Romero-Romo

**Affiliations:** Graduated Studies in Science and Engineering of Materials, Universidad Autónoma Metropolitana-Azcapotzalco, Ave. San Pablo 180, Col Reynosa CP 02200, Mexico; trm@correo.azc.uam.mx (M.T.-R.); gam@correo.azc.uam.mx (M.G.-A.); mmrr@correo.azc.uam.mx (M.R.-R.)

**Keywords:** hybrid adhesive, aluminum substrate, sol-gel treatment, shear strength, zirconia nanoparticles, silica nanoparticles

## Abstract

This research presents the interaction of the epoxy polymer diglicydil ether of bisphenol-A (DGEBA) with silica (SiO_2_) nanoparticles plus zirconia (ZrO_2_) nanoparticles obtained via the sol-gel method in the synthesis of an epoxy-silica-zirconia hybrid adhesive cured with polyamide. ZrO_2_ nanoparticles were added to the epoxy-silica hybrid adhesive produced in situ to modify the apparent shear strength of two adhesively bonded aluminum specimens. The results showed that the addition of different amounts of ZrO_2_ nanoparticles increased the shear strength of the adhesively bonded aluminum joint, previously treated by sandblasting, immersion in hot water and silanized with a solution of hydrolyzed 3-glycidoxipropyltrimethoxysilane (GPTMS). The morphology and microstructure of the nanoparticles and aluminum surfaces were examined by scanning electron microscopy (SEM), and elemental analysis was performed with the Energy-dispersive X-ray spectroscopy (EDS) detector; the chemical groups were investigated during the aluminum surface modification using Fourier transform infrared spectroscopy (FTIR).

## 1. Introduction

Epoxy adhesives have been used for many years in a great number of engineering applications, e.g., aerospace, marine and automotive industries, where high durability bonded aluminum structures are required [1,2]. However, it is well known that epoxy organic materials cannot be used in high performance applications due to their limited mechanical, chemical and thermal properties.

Therefore, a systematic investigation of both the hybrid materials synthesis and mechanical response is an important issue to improve those properties [3].

Thermosetting epoxy polymers are widely used as adhesives, they are amorphous and highly-crosslinked, and the microstructure of these materials has many interesting properties for structural engineering applications, such as a high modulus, high failure strength, low creep and interesting adhesive properties. Nevertheless, the structure of such thermosetting polymers can also lead to an undesirable high brittleness, with poor resistance to crack initiation and growth [4].

The epoxy-silica hybrid system is one of the most recognized composites in the field of adhesives, since in addition to the excellent adhesive properties of cured epoxy polymers, the silica nanoparticles, or nanofillers strengthen the epoxy resin, improving the toughness of the organic matrix [5].

The toughening mechanism of organic polymers has been explored extensively; when solid particles are incorporated in the epoxy matrix they enhance the epoxy’s fracture toughness through different mechanisms of energy dissipation. Solid particles, whose adhesion strength to the epoxy matrix is greater than the stress or tension to which the hybrid adhesive is subjected, interfere with the crack growth via pinning, branching, bridging and/or deflection to reduce the crack-driving force at the crack tip. Conversely, particles with poor adhesion to the organic matrix debond easily to activate different energy dissipation mechanisms like diffused matrix shear yielding or plastic void growth [6].

When aluminum–aluminum substrates are adhesively bonded with epoxy-silica hybrid adhesives, some of the negative effects that can arise from this process are: (a) the viscosity of the polymer mixture increases dramatically and will be too high to process, this happens when there are high loading nanofillers and/or when the nanofiller size becomes smaller [3]; (b) the formation of nanofiller agglomerates that decrease the dispersion of the adhesive on the substrate [7]; and (c) another possible problem during the adhesion of aluminum-aluminum specimens is due to contraction occurring in the organic matrix during polymerization, mostly caused by the viscous-elastic properties producing contraction stresses, which can damage the adhesive interface and potentially produce debonding [8]. A possible solution to the limitations created by the inclusion of a single type of nanoparticle in the epoxy resin (agglomeration, viscosity increase and matrix contraction) is the addition of two or more sorts of nanoparticles, in addition to SiO_2_ nanoparticles, such as clay, alumina, titania or zirconia as fillers in epoxy resins. This produces a synergic effect since the combination of two nanomaterials brings greater strength to the adhesive capacity than what can be attained by a single nanomaterial [6].

Only a few studies of epoxy-ZrO_2_ composites related to their morphology and mechanical properties have been reported [9], and they show that the mechanical resistance and toughness of an epoxy resin are improved by increasing the content of the ZrO_2_ nanoparticles, This is attributed to the fact that the ZrO_2_ has a much higher strength than the epoxy matrix alone, and to the good bonding between the filler and the matrix. Behzaddnasab et al., obtained higher coating toughness with the addition of aminopropyltrimethoxy silane (APS) to treated zirconia nanoparticles in an epoxy based coating [10]. Afterwards, the same group studied the effect of the addition of different combinations of clay and APS-treated zirconia as reinforcement to organic polymers increasing the corrosion performance compared with neat-epoxy coating, via enhancing the barrier properties and ohmic resistance [11,12]. Regarding epoxy adhesives, the dispersion of ZrO_2_ nanoparticles into commercial epoxy resin commonly used for the preparation of structural adhesives, using an amine as a hardener, showed mechanical performance improvements [13].

The pretreatment of aluminum surfaces is one of the most important stages in adhesion. Regardless of the treatment used, the surface of the substrate is modified basically by: (a) increases in surface tension; (b) increases in surface contact; (c) surface substrate chemical modification, or a combination of the previous modifications [14]. Critchlow and Brewis reported a summary of the most common surface treatments for aluminum specimens for bonding [15]. However, Rider [16] and Johnsen et al. [17] reported a treatment for aluminum specimens using three different steps, grit-blasting, boiling water and silanization. Abel et al., used the silanization technique to show that the covalent Al-O-Si bond is formed between hydrolyzed 3-glycidoxipropyltrimethoxysilane (GPTMS) and aluminum surfaces [18].

The present study is concerned with the effects on the bonding performance of two aluminum specimens due to the addition of different ZrO_2_ nanoparticle masses as a second nanoparticle filler in several epoxy-silica hybrid adhesives, before the curing process with polyamide. Furthermore, the effect of different physical and/or chemical pretreatments on the aluminum surface was assessed.

## 2. Results and Discussion

### 2.1. Epoxy-Silica Hybrid Adhesives

**Figure 1:**
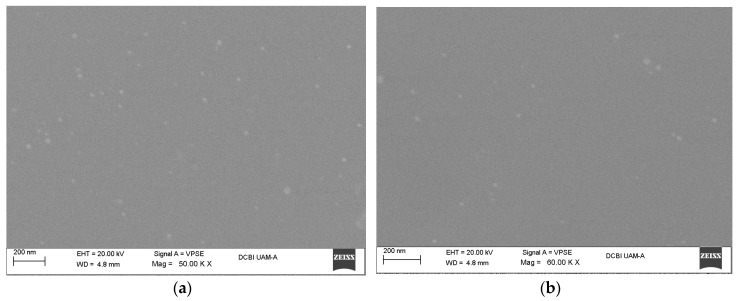
SEM micrographs of the silica nanoparticle dispersion with a 0.75 PHR (part of SiO_2_ per hundred parts of resin) content, employing the sol-gel in situ technique: (**a**) Tetraethyl orthosilicate (TEOS) precursor; (**b**) 3-glycidoxipropyltrimethoxysilane (GPTMS) precursor.

Figure 1 shows the SEM micrographs of the epoxy-silica hybrid material prepared at 0.75 PHR (part of SiO_2_ per hundred parts of resin) employing a sol-gel in situ technique using tetraethyl orthosilicate (TEOS) and GPTMS as precursors. It is possible to observe, in contrast respect to the gray homogeneous background, some lighter objects that correspond to silica nanoparticles, which were dispersed and embedded in the epoxy matrix; they form an interpenetrating network structure that stands out from the background, with a particle size of up to 50 nm in the organic matrix.

**Figure 2:**
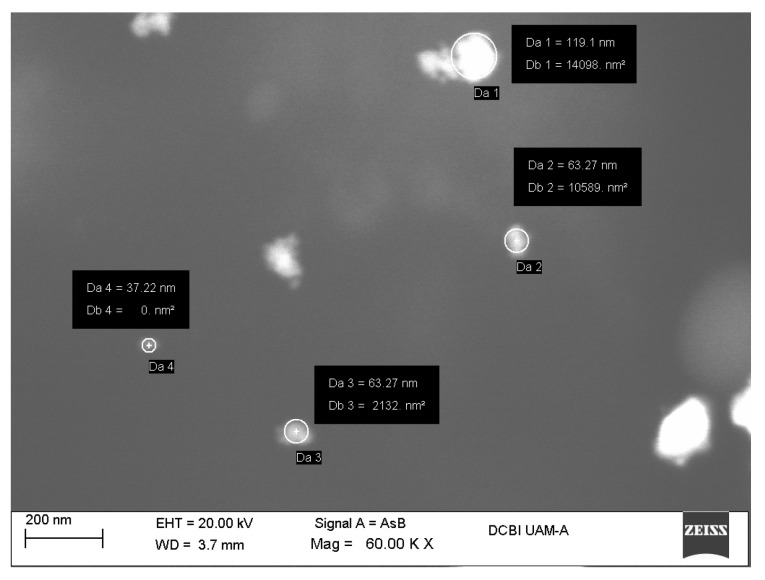
SEM micrographs of zirconia nanoparticles synthesized employing the sol-gel technique.

Figure 2 shows analysis by a backscattered electron image of the zirconia nanoparticles synthesized through the sol-gel process, with sizes from 30 nm to 80 nm, which are not homogeneously shaped and appear to have formed some nanoparticle aggregates dispersed in the polymer matrix [19].

**Figure 3:**
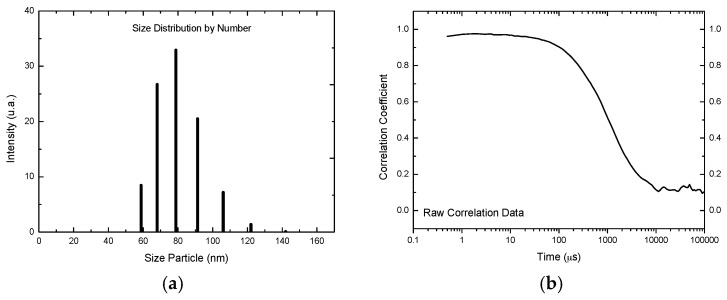
(**a**) Zirconia nanoparticles size distribution; (**b**) Raw correlation coefficient.

Figure 3a shows the size distribution by the number of zirconia nanoparticles used as a second filler in the epoxy-silica hybrid adhesive, which corroborates the results presented in Figure 2. Figure 3b presents the correlation coefficient displayed by the Zetasizer Nano equipment ZS90 (Malvern Instruments Ltd, Worcestershire, UK).

### 2.2. Rheology of Epoxy-Silica Hybrid Adhesives

**Figure 4:**
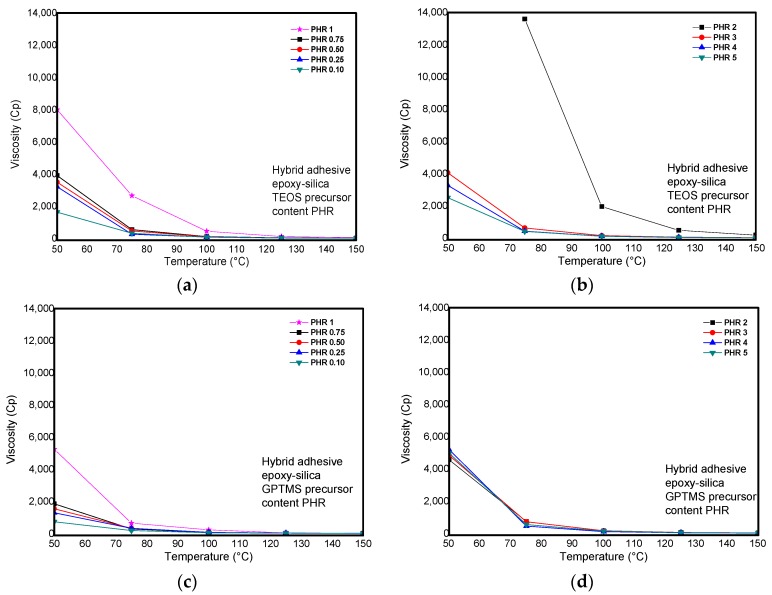
Viscosity of the epoxy-sílica matrix synthesized in situ varying the content of different inorganic precursors: (**a**,**b**) TEOS precursor; (**c**,**d**) GPTMS precursor.

Figure 4 shows the effect on the viscosity of the epoxy-silica hybrid adhesive, called class I and class II, by different silica nanoparticles, evaluated at 50 °C, 75 °C, 100 °C, 125 °C and 150 °C. It can be observed that at low temperature (50 °C) and nanoparticle content of 1 PHR that viscosity increased drastically becoming the epoxy-silica hybrid highly unmanageable, whereas for a silica nanoparticle content above 2 PHR, a viscosity decrease was observed because of the agglomeration and precipitation of nanoparticles.

This behavior is more relevant when the TEOS was used as precursor, which shows an important physical interaction with the epoxy matrix. Meanwhile, when GPTMS was employed it seems that the functional group present in GPTMS has a lesser physical interaction.

Good spreading or wetting is required to ensure high interfacial contact between the two phases when applying a liquid polymeric adhesive on a rigid substrate. The spreading or wetting mechanism is influenced by both the viscosity value of the adhesive hybrid as well as the uncompensate surface tension forces. Both are two highly important characteristic properties of an adhesive joint, consistent with those reported by Paz et al. [20], and Carré & Eustache [21].

### 2.3. Aluminum Physical Treatment

**Figure 5:**
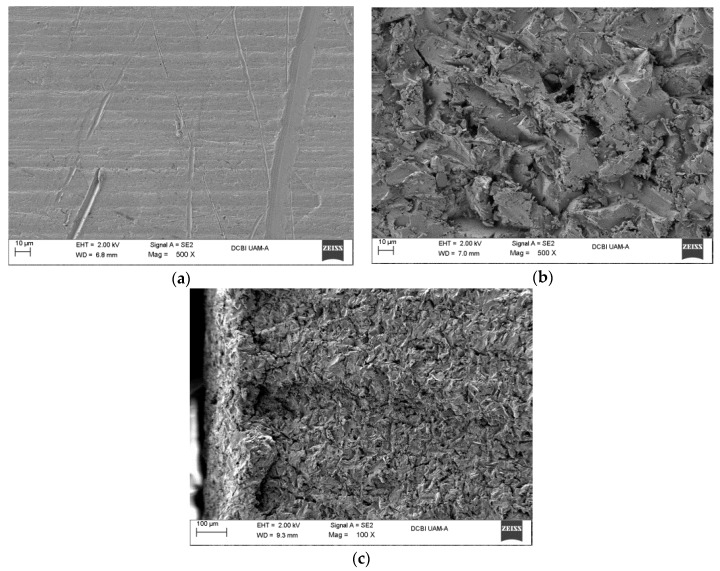
Surface topological features of sandblasted aluminum surfaces: (**a**) Top view of a commercial aluminum at 500×; (**b**) Top view of a commercial aluminum with sandblasting at 500×; (**c**) Lateral view of sandblasting at 100×.

Prior to the bonding procedure, the aluminum substrate surfaces were treated by different physical and chemical procedures. Figure 5a shows the commercial aluminum surface specimen; Figure 5b shows a specimen treated with sandblasting, where the aluminum sample’s surface morphology was considerably changed after treatment, producing sharp crests due to the high-speed impacts of 50 μm size silicon carbide particles; also there appears numerous irregular surfaces, which improve the wetting of adhesives on solid substrates [20,22]. Figure 5c shows the surface topography in a lateral view of the aluminum specimen, the roughness is due to sandblasting and its approximate depth is around 60 μm.

The elemental analysis from the SEM/EDS of the surface of the commercial aluminum specimen and a sandblasted aluminum specimen can be seen in Table 1. It is important to note the different oxygen contents between the specimens; aluminum, carbon, and silicon, due to the silicon carbide from the sandblasting process.

### 2.4. Aluminum Hydrotermal Treatment at 85 °C

Table 2 presents the weight increase of the aluminum specimens immersed in deionized water at 85 °C for different times, according to the reaction below [23]; the initial specimen weight was 0.1314 g.

2Al + 5.1H_2_O → Al_2_O_3_·2.1H_2_O + 3H_2_

Such weight increase is due to the aluminum dissolution-precipitation phenomena which has a high initial velocity followed by a decay period [22].

**Figure 6:**
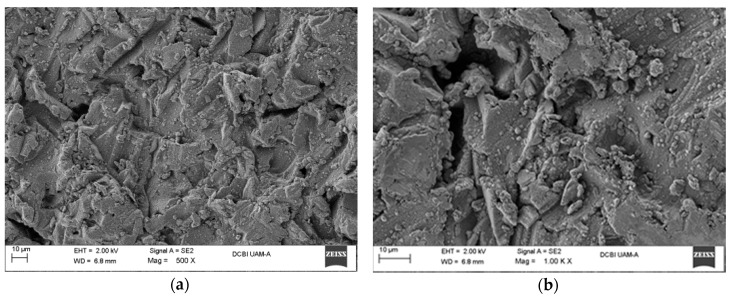

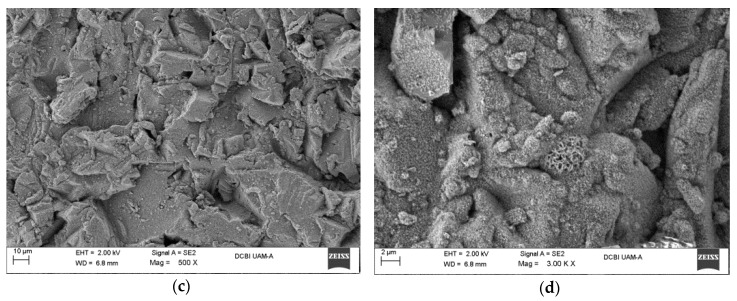
SEM micrographs of aluminum specimens after immersion treatment in water at 85 °C for 20 min, at different magnifications: (**a**) 500×; (**b**) 1 K×, and 60 min treatment; (**c**) 500×; (**d**) 3 K×.

Figure 6 presents the topography of aluminum specimens taken at two different immersion times. It is possible to observe a surface roughness change that causes an increase in surface area, at 20 min in Figure 6a,b and at 60 min in Figure 6c,d; so, the surface morphology changes with increasing immersion time in hot deionized water [22]. The rapid growth stage involves a dissolution-precipitation mechanism and a final slow growth stage occurs, perhaps because the film density increases, as a consequence of a slower migration of soluble species to the film-solution interface.

Table 3 displays the EDS analysis of the aluminum substrate immersed in deionized water at 85 °C where the oxygen content increased with immersion time. The formation of a pseudobohemite layer with some gibbsite and boyerite indicate changes in the chemical composition and roughness of the surface and this improves the adhesive bonds [22]; these results are consistent with Rider and Arnot who treated aluminum with hot water [24].

**Figure 7:**
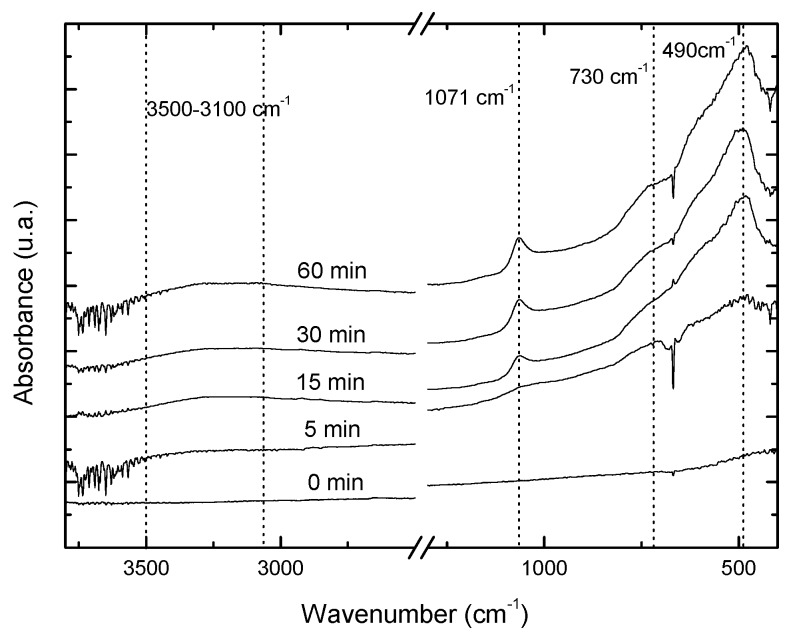
Fourier transform infrared spectroscopy (FTIR) of the film produced on aluminum immersed in water at 85 °C as a function of time.

Figure 7 shows the Fourier transform infrared (FTIR) spectrum of the film formed on aluminum immersed in hot deionized water at different times; in all cases a peak is present at the 730 cm^−1^ band due to the Al-O bond vibration that increases with time, which is associated with boehmite and pseudoboehmite. The peak at the 1071 cm^−1^ band is due to the δ OH characteristic of pseudoboehmite, and a very wide peak in the band from 3100 cm^−1^ to 3500 cm^−1^ appears to be associated with the development of pseudoboehmite; these results corroborate that the aluminum specimens immersed in deionized water at 85 °C formed a pseudoboehmite layer on the surface.

### 2.5. Silanization Treatment of Aluminum Specimen with GPTMS

Before silanization of the aluminum specimen it is necessary to hydrolyze the GPTMS in order to produce the silanols groups. It is important to avoid the condensation process of such silanols at this stage: the hydrolysis and condensation have an important effect in the adhesive process because high levels of condensation are unfavorable for adhesion due to the loss of silanol groups, which promote covalent bonds with surface aluminum oxides during adhesion.

Table 4 presents the weight change of the aluminum substrates immersed in hydrolyzed 3% GPTMS aqueous solution at different times. The initial specimen weight was 0.2897 g.

**Figure 8:**
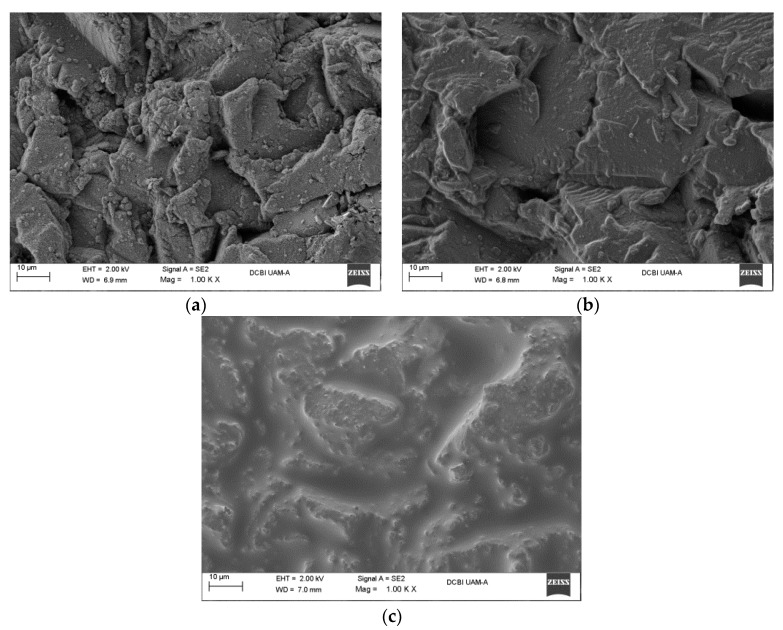
SEM micrographs of aluminum specimens after immersion in 3% hydrolyzed GPTMS solution at 25 °C, at 1 K×, (**a**) 10 min; (**b**) 20 min; (**c**) 60 min.

Figure 8 shows micrographs of the morphology of different aluminum specimens which were immersed in a hydrolyzed GPTMS aqueous solution; Figure 8a depicts the aluminum substrate after 10 min immersion; Figure 8b shows the aluminum substrate after 20 min immersion, and Figure 8c portrays the aluminum substrate immersed for 60 min.

The aluminum specimen immersed for 20 min developed a thin homogeneous coating layer distributed over the aluminum surface, meanwhile after 60 min immersion, the coating layer thickened in the valleys as shown in Figure 8. Thus, the previous outcome affirms that treatment with hydrolyzed GPTMS is a factor that influences the quality of the Si-O-Al bonds formed on the aluminum surface [25].

Table 5 displays the EDS analysis of aluminum substrates after sandblasting, followed by water immersion at 85 °C plus silanization treatment; the decrease in the oxygen content is due to the formation of water during the condensation process because of the formation of Al-O and Si-O covalent bonds on the aluminum surface and the subsequent evaporation process. Carbon is present due to the GPTMS molecule; lastly there is an increase in the silicon content because of the condensation process during silanization.

**Figure 9:**
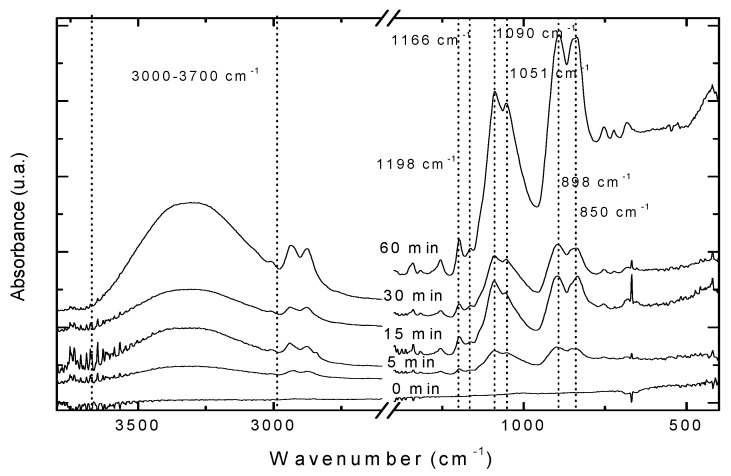
FTIR of the film produced on aluminum specimen silanized with 3% GPTMS at different times.

The FTIR spectrum in Figure 9 reveals the behavior of the aluminum substrates surface after different immersion times in the 3% hydrolyzed GPTMS solution. It was observed that the epoxy ring in the 898 cm^−1^ band attributed to epoxy group, increases with time, with the same behavior in the 850 cm^−1^ band, this is attributed to methoxy groups. The peaks in the 1000–1250 cm^−1^ absorption bands are attributed to Si-O-Si vibration [17]. The peak in the 3000–3370 cm^−1^ band is associated with silanol groups and the peak centered in the 1051 cm^−1^ band is associated with Si-O-Al bonding [26].

### 2.6. Drying Aluminum Treatment

**Figure 10:**
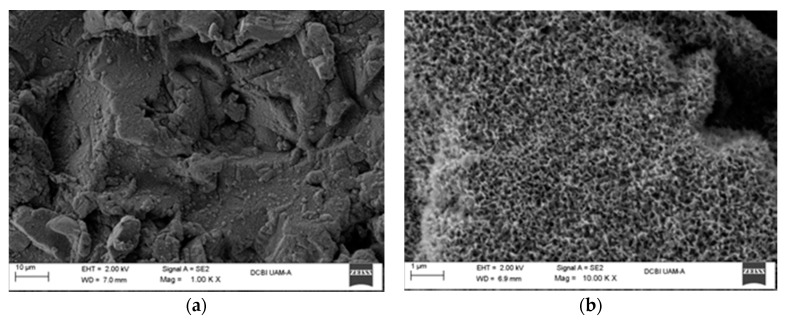
SEM micrographs of aluminum specimens dried for 60 min at 100 °C, (**a**) 1 K×; (**b**) 10 K×.

SEM micrographs in Figure 10 show the aluminum specimens after these were dried for 60 min at 100 °C. An inorganic, highly rough film can be seen in Figure 10b.

**Figure 11:**
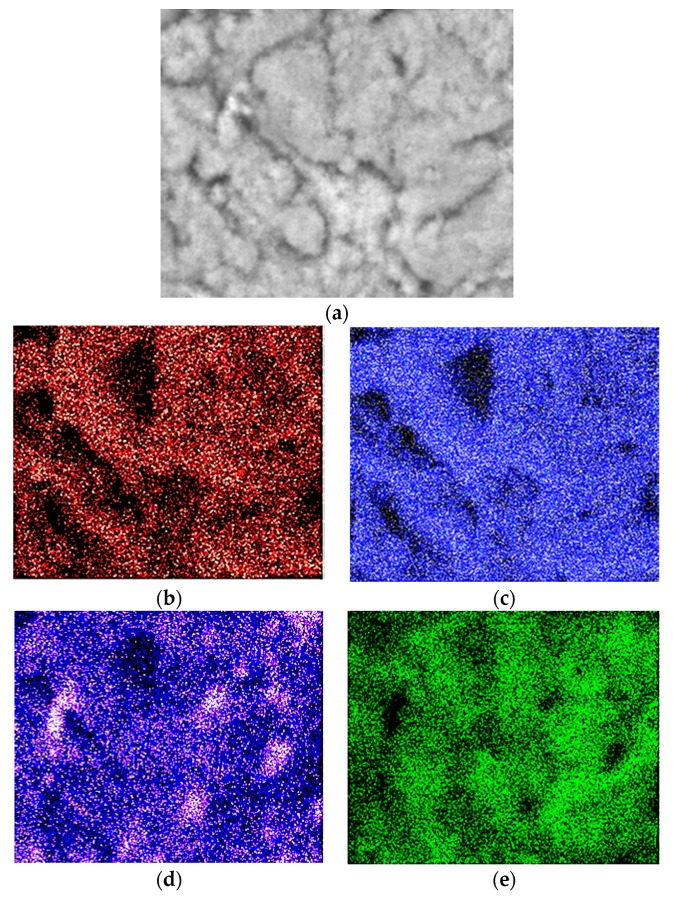
(**a**) SEM micrograph of aluminum specimen dried for 60 min at 100 °C, at 5 K×; (**b**) EDS mapping of C; (**c**) EDS mapping of O; (**d**) EDS mapping of Si; (**e**) EDS mapping of Al.

Figure 11 shows the SEM and EDS mapping of the elements at 5 K× magnification: the micrograph presents a homogeneous dispersion of carbon (b), oxygen (c), and silicon (e) over the aluminum surface (d) after the drying process.

Table 6 displays the EDS analysis of the aluminum specimens after drying; it is observed that the oxygen and silicon contents have increased while the carbon content remains almost constant.

**Figure 12:**
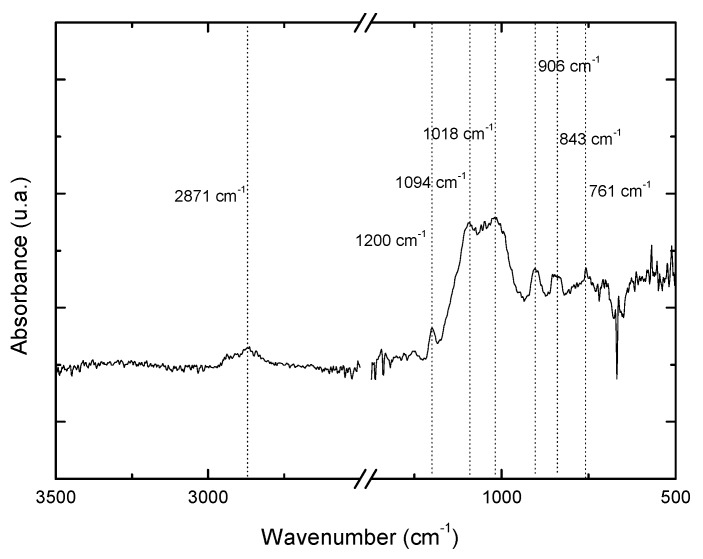
FTIR of the film produced on the aluminum specimen silanized with 3% GPTMS after drying.

The FTIR spectrum in Figure 12 reveals the behavior of the aluminum specimen surface after drying. It is observed that the epoxy ring in the 908 cm^−1^ and the 840 cm^−1^ bands are attributed to methoxy groups, which are very small compared to the same absorption bands shown in Figure 9; the absorption band at 3550 cm^−1^ indicates that the silanol groups formed the covalent bonds Si–O–Si and Si–O–Al during the condensation process, and the water from the condensation reaction has evaporated during drying. The absorption in the 1000–1250 cm^−1^ band is attributed to Si–O–Si vibration [1,17] and the 1018 cm^−1^ absorption band is related to the asymmetric Si-O-Al stretching mode [26,27].

### 2.7. Shear Strength Testing of Adhesively Bonded Single Lap Joints

**Figure 13:**
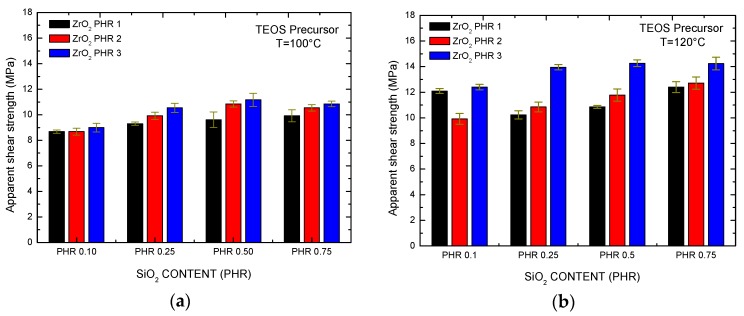
Apparent shear strength of epoxy-SiO_2_-ZrO_2_ adhesives using TEOS as a precursor at different temperatures: (**a**) T = 100 °C; (**b**) T = 120 °C.

Figure 13 shows the apparent shear strength test for an adhesive joint of two aluminum specimens with an epoxy-SiO_2_-ZrO_2_ hybrid adhesive with a low content of silica nanoparticles measured as parts per hundred of resin, PHR (silica was synthesized with TEOS precursor), and cured with polyamide at 100 °C and 120 °C.

It was observed that for both temperatures the zirconia PHR content modified the shear strength, and the best results obtained were those with 0.75 PHR silica and 3 PHR zirconia nanoparticles at 120 °C. The improved toughness effect due to the ZrO_2_ nanoparticles was propitiated by their interaction with the organic matrix, encouraging better energy dissipation mechanisms. Johnsen et al. [4], concluded that the increase in shear strength in the hybrid material is caused by nanoparticles debonding followed by plastic void growth, as the energy dissipation mechanism.

**Figure 14:**
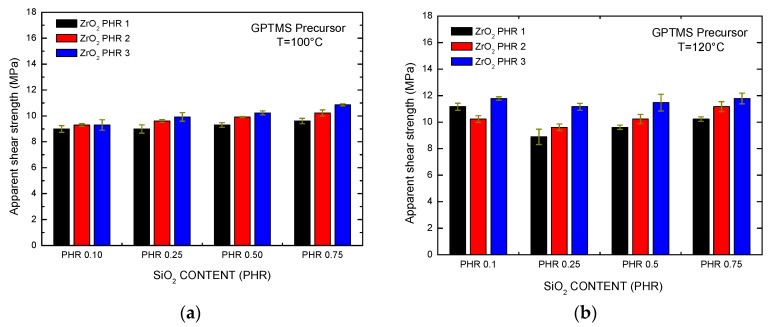
Apparent shear strength of epoxy-SiO_2_-ZrO_2_ adhesives using the GPTMS precursor at different temperatures: (**a**) T = 100 °C; (**b**) T = 120 °C.

Figure 14 shows the apparent shear strength test for an adhesive joint of two aluminum specimens with an epoxy-SiO_2_-ZrO_2_ hybrid adhesive with low PHR silica content, cured with polyamide at 100 °C and 120 °C; in this case, the adhesive epoxy-silica was synthesized in situ with the GPTMS precursor, the most important chemical feature is to have an oxyrane group in the GPTMS molecule, promoting the generation of covalent bonds with the polyamide. The best results were obtained with the highest zirconia content at PHR 3; although, in general, all apparent shear strength values of these hybrid adhesives were lower than those obtained with the TEOS precursor, suggesting that the hydrolysis of the GPTMS methoxy groups is not fast enough to produce hydroxyl groups, since instead of this, the formation of methoxylated oligomers takes place influencing the kinetics of the silicon-network formation [28].

**Figure 15:**
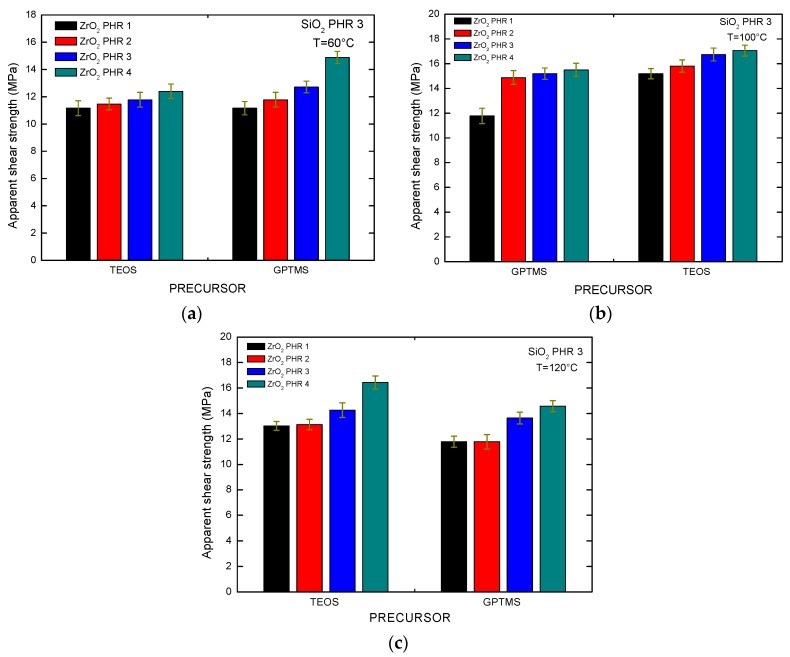
Apparent shear strength for epoxy-silica-zirconia adhesives with TEOS and GPTMS precursors at different temperatures: (**a**) T = 60 °C; (**b**) T = 100 °C; (**c**) T = 120 °C.

Figure 15 shows the results of shear strength testing of epoxy-silica hybrid (TEOS and GPTMS) adhesives with 3 PHR silica nanoparticles and different contents of zirconia nanoparticles, at three polyamide curing temperatures, namely: 60 °C, 100 °C and 120 °C. In general, all tests display high shear strength for the TEOS-synthesized silica compared with the GPTMS precursor. Figure 15 displays the best shear strength performance of the hybrid adhesives, at the highest zirconia content of PHR 4. Although the highest shear strength was observed at 100 °C; those at 60 °C depicted the presence of a soft adhesive, whereas that cured at 120 °C turned out to be fragile. It is concluded that the optimum temperature of reaction must be selected between the competing factors of viscosity decrease due to temperature, and the immobility of polymeric chain increase due to reaction. Both factors can be balanced successfully to produce the desired properties in the thermosetting hybrid adhesive [29].

**Figure 16:**
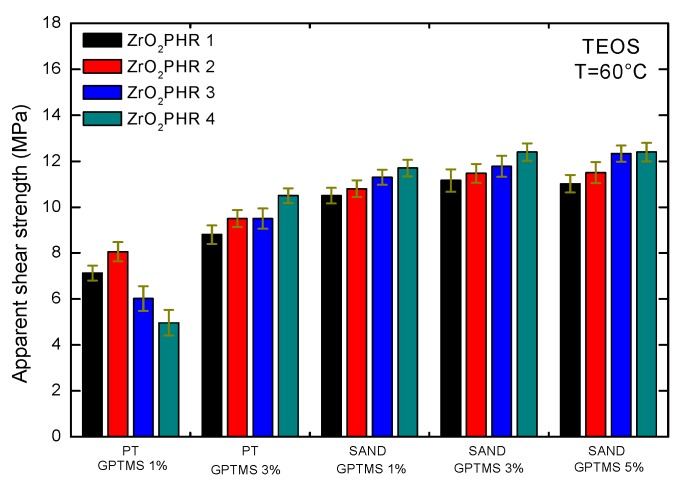
Apparent shear strength with different surface treatments of aluminum specimens.

Figure 16 presents the apparent shear strength tests with different surface treatments of the aluminum specimens. The two first groups of tests correspond to physical treatment followed by immersion in hot water and silanization in an aqueous solution with hydrolyzed GPTMS at 1% and 3%. The other three groups of specimens were treated with sandblasting plus immersion in hot water and silanization in aqueous solution with hydrolyzed GPTMS at 1%, 3% and 5%. All aluminum specimens were adhesively bonded utilizing polyamide as a curing agent at 60 °C; the best shear strength results were obtained with 4 PHR ZrO_2_ content and sandblasting with hot water plus silanization in 3% hydrolyzed GPTMS.

### 2.8. Fracture Analysis

**Figure 17:**
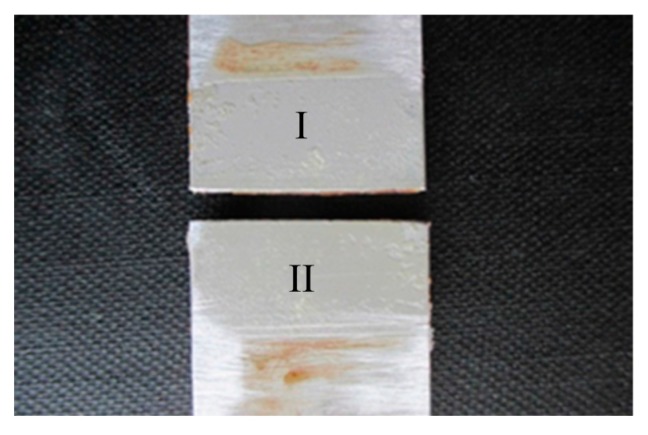
Aluminum–aluminum specimens after the shear strength test. (Specimen I and specimen II).

Figure 17 shows two aluminum specimens of the assembly joint (specimen I and specimen II) after the apparent shear strength test where it can be observed that the rupture of the joint happened in the adhesive itself, thus demonstrating the great adhesive strength of the aluminum–aluminum assembly.

**Figure 18:**
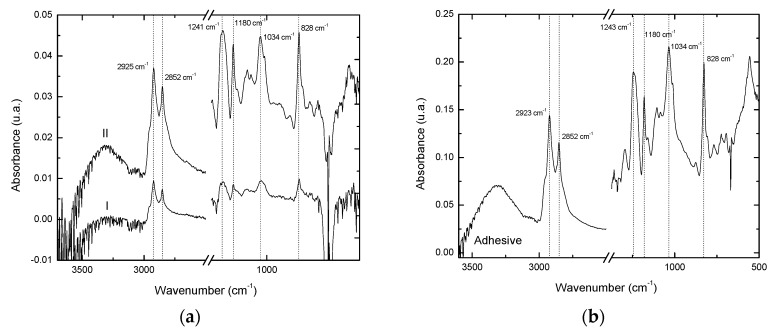
FTIR spectra: (**a**) From both faces I and II of the adhesive bond after the shear strength test; (**b**) From the cured hybrid adhesive.

Figure 18, shows that the FTIR spectrum from both aluminum specimen surfaces I and II, and the spectra of the cured hybrid adhesive have the same absorbance spectra in common, from which it can be concluded that the fracture of the joint occurred in the adhesive matter itself, as mentioned above.

**Figure 19:**
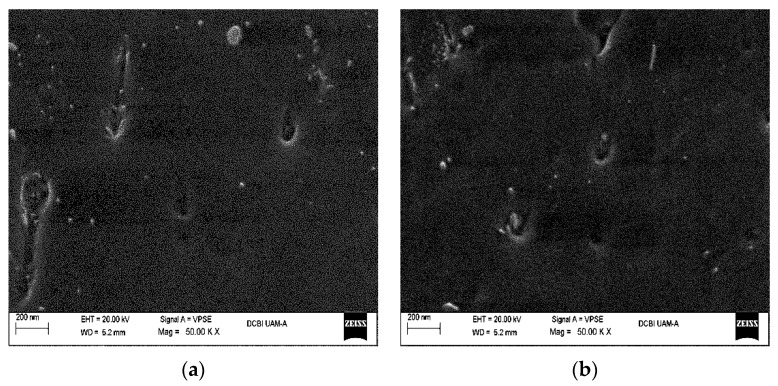
(**a**,**b**) SEM micrographs of the hybrid material, show the deformation of the organic matrix due to the energy dissipation at 50 K×.

The micrographs (a) and (b) shown in Figure 19 depict the effect of silica nanoparticles on the shear stress line and the deformation of the organic matrix due to energy dissipation during separation of both the hybrid adhesive and the substrate; the debonding effect plus matrix shear yielding or plastic void growth [6] can be clearly observed.

## 3. Materials and Methods

### 3.1. Synthesis of Hybrid Adhesives of Inorganic-Organic Composition

Silica based inorganic-organic hybrid materials were prepared in situ by the sol-gel method admixing commercial liquid DGEBA (standard YD-128, from Epoxemex S.A. de C.V. (Mexico City, Mexico)) deionized water, ammonium hydroxide and ethyl alcohol with two different silica precursors. In the first case, tetraethyl orthosilicate (TEOS) 99.99%, labeled (class I) was used and in the second case, 3-glycidyloxypropyltrimethoxysilane (GPTMS) ≥98%, labeled (class II), both in a H_2_O to 6 Si mass ratio and H_2_O equal to EtOH mass ratio, at pH 9 using NH_4_(OH) [30], and in both cases nanoparticles were prepared in situ to obtain hybrid adhesives with different nanoparticles mass ratio: PHR 5, 4, 3, 2, 1, 0.75, 0.50, 0.25, 0.10; where PHR is defined as one part of SiO_2_ per hundred of resin; all reactants were from Sigma-Aldrich (St. Louis, MO, USA).

### 3.2. Synthesis of ZrO_2_ Nanoparticles and the Epoxy-SiO_2_-ZrO_2_ Hybrid Adhesive

The synthesis of zirconia nanoparticles was conducted by the sol-gel technique in a glove box using as a precursor, zirconium-propoxide solution 70% in 1-propyl alcohol, deionized water, anhydrous propyl alcohol and nitric acid (70%); all reactants were from Sigma-Aldrich, in a ratio of zirconium reference of 1:1.5:12:0.3, respectively.

During synthesis, a solution of a definite amount of zirconium-propoxide in an anhydrous propyl alcohol and nitric acid was prepared in an Erlenmeyer flask maintaining vigorous agitation using a magnetic stirrer; the temperature was maintained at 86 °C for one hour with strong stirring, then a solution of a definite amount of water and propyl alcohol was added.

Nanoparticles were filtered and washed with deionized water, then calcined in a tubular oven (mod 21100, Thermolyne tube furnace^®^ (Sigma-Aldritch, St. Louis, MO, USA) at 500 °C for 4 h, a monoclinic phase of ZrO_2_ was obtained, the density was 5.7 g·cm^−3^ picnometry, and the isoelectric point was at a pH of 5.2, which is in agreement with other authors [31,32,33]. ZrO_2_ nanoparticles were mixed with the hybrid epoxy-silica system at several PHR (1, 2, 3, 4) to obtain the epoxy-silica-zirconia hybrid adhesive and then cured with polyamide from Epoxemex S.A. de C.V.

**Scheme 1:**
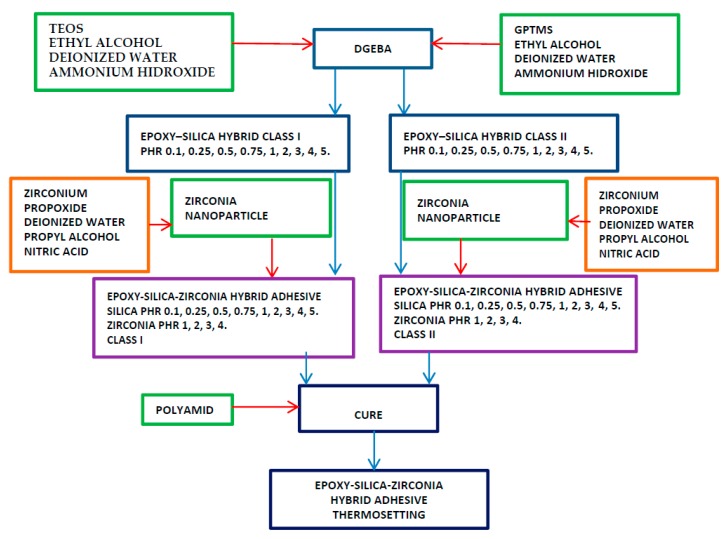
Methodology for the synthesis of the epoxy-silica-zirconia hybrid adhesive.

Scheme 1 expounds the methodology used to synthesize the epoxy-silica-zirconia hybrid adhesive, before its application to the aluminum specimens with different pretreatments.

### 3.3. Treatment of Aluminum Substrates

**Figure 20:**
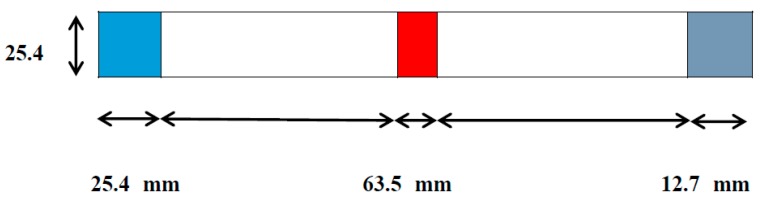
Aluminum specimen of ASTM STANDARD D1002-05.

The substrates used were two aluminum specimens according to ASTM D1002-05 [34], the dimensions and geometry of the aluminum specimens are shown in Figure 20.

Each substrate was 101.6 mm in length, 25.4 mm wide, with a thickness of 1.62 mm, and the overlap length of the substrates was 12.7 mm, corresponding to an adhesion surface of 322.58 mm^2^.

All aluminum samples were subjected to a water-rinsing step followed by washing with deionized water and acetone. Table 7 presents the different surface treatments applied to the aluminum specimens before the application of the epoxy-silica zirconia hybrid adhesives.

### 3.4. Instrumental Analysis

The morphology and microstructure of the nanoparticles and the surface of the aluminum specimens were examined by using SEM (Carl Zeiss, model Supra 55VP (Carl Zeiss, Oberkochen, Germany)). Samples were attached to the sample holder with standard carbon adhesive tabs (Electron Microscopy Sciences (Hatfield, PA, USA)) and analyzed in variable pressure modes within the 2 kV to 20 kV beam energy range. Elemental analyses were performed with an EDS detector (Oxford Instruments, Abingdom, UK).

Infrared absorption of the small samples of adhesives in the 4000 cm^−1^ to 350 cm^−1^ range was analyzed by Fourier transform infrared spectroscopy (FTIR) (Varian, Palo Alto, CA, USA) with a Varian model Excalibur 3600. The equipment has a DTGS detector, beam splitter (KBr) and a device of attenuated total reflectance with a 4 cm^−1^ resolution, with a diamond plate for non-destructive analysis (PIKE Technologies Gladiat ATR™, Madison, WI, USA).

The viscosity of the epoxy-silica hybrid adhesive class I and class II with different nanoparticle content was assessed with a rotational Anton Paar rheometer model MCR-502 (Anton Paar GmbH, Graz, Austria), a parallel plate geometry was used, with a shear rate controlled rotational test with 8 viscosity testing points at 50 °C, 75 °C, 100 °C, 125 °C and 150 °C.

Size distribution of ZrO_2_ nanoparticles was assessed with a Zetasizer Malvern 90 series equipment (Malvern Instruments Ltd, Worcestershire, UK) using a 90 degree scattering angle using Dynamic Light.

### 3.5. Mechanical Testing

**Figure 21:**
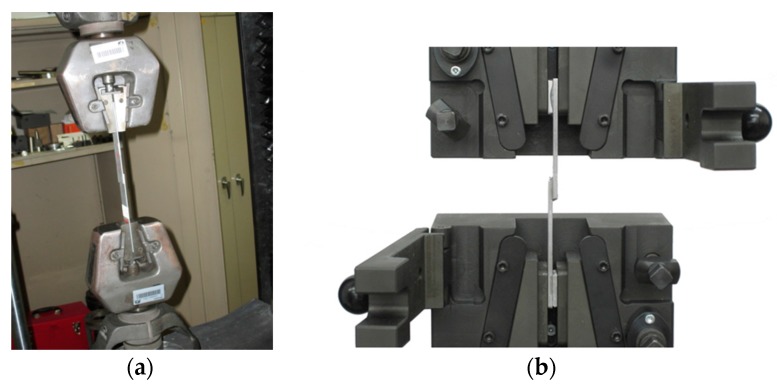
(**a**) Photograph of grips employed and set up of the Instron model 1125; (**b**) Cross section of the supplier’s grips.

Shear strength tests were performed at least in triplicate using an Instron testing machine model 1125 (Instron^®^, Norwood, MA, USA) according to the Standard Test ASTM D1002-05. Figure 21 shows the aluminum specimen employed for the apparent shear strength in the test method.

Shear testing of a single-lap measures the maximum shear stress that may be sustained by a material before it breaks. A single-lap joint is a geometry very frequently employed to adhesively join two aluminium specimens because it is the correct technique to join them and it is the standard test method for evaluating the apparent shear strength of an adhesive bond, which is the basis for adhesive joint design [35].

## 4. Conclusions

The results of the shear strength test carried out on the aluminum specimens bonded adhesively shows, in the great majority of the assays, that there is indeed an increase in toughness corresponding with the increase in the zirconia content. Therefore, under the conditions that prevail in the preparation of the hybrid adhesive and the pretreatments of the aluminum specimens, there is synergy between the two classes of nanoparticles employed.

When TEOS was the precursor for the synthesis of the silica nanoparticles of the hybrid adhesive, the shear strength was generally greater compared with the hybrid adhesive prepared with GPTMS, meaning that there is more effective energy dissipation in the bonding created for the silica synthesized with a non-functionalized precursor than with the functionalized one.

The aluminum sandblasting surface treatment, with hydric treatment plus silanization and 3% GPTMS presented the best results for shear strength. This implies that in these conditions there was more Si-O-Al covalent bonding.

## Figures and Tables

**Table 1:** EDS analysis of the commercial and sandblasting aluminum substrate surfaces.

Aluminum Specimen Type	Wt % O	Wt % Al	Wt % C	Wt % Si	Wt % Other
Commercial aluminum	6.90	68.76	22.97	0.44	0.93
Sandblasted aluminum	5.71	76.15	8.73	8.57	0.59

**Table 2:** Weight increase of the pseudoboehmite layer after increasing immersion times in water at 85 °C.

Immersion Time (min)	Final Weight (g)	Weight Increase %
5	0.1316	0.1522
15	0.1317	0.2432
30	0.1322	0.6088
60	0.1325	0.8371

**Table 3:** EDS analysis of the aluminum substrate surface treated with hot water.

Immersion Time (min)	Wt % O	Wt % Al	Wt % C	Wt % Si	Wt % Other
0	5.71	76.40	8.73	8.57	0.59
20	16.23	70.03	7.48	4.8	1.46
60	24.82	62.58	6.80	4.60	1.20

**Table 4:** Weight increase of aluminum specimens after immersion in hydrolyzed 3% GPTMS at pH 5 and 25 °C.

Immersion Time in GPTMS (min)	Final Weight (g)	Weight Increase %
5	0.2912	0.5177
15	0.2914	0.5868
30	0.2915	0.6213
60	0.2917	0.6903

**Table 5:** EDS analysis of the aluminum substrate surface.

Immersion Time (min)	Wt % O	Wt % Al	Wt % C	Wt % Si	Wt % Other
0	16.23	70.03	7.48	4.80	1.46
20	11.30	58.42	18.30	11.18	0.80
60	12.38	50.28	20.20	16.40	0.74

**Table 6:** EDS analysis of aluminum specimen after the drying process.

Aluminum Specimen	Wt % O	Wt % Al	Wt % C	Wt % Si	Wt % Other
Before drying	11.30	58.42	18.30	11.18	0.80
After drying	17.38	47.50	19.00	15.06	1.06

**Table 7:** Surface pretreatment for aluminum specimens.

Treatment	Description
Physical treatment	Abrasion with Scotchbrite fiber under rinsing water.
Physical + Chemical treatment with hot water (CTHW)	Immersion in water at 80 °C without stirring to allow a boehmite layer AlO(OH) to be formed, at different times.
Physical + CTHW + silanization	GPTMS was hydrolyzed in water at 25 °C for 60 min, then the aluminum specimen was immersed for 10 min at 25 °C and dried in a stove at 100 °C, for 60 min.
Sandblasting + CTHW + silanization	The aluminum specimen was sandblasted with silicon carbide (particles of 50 μm diameter) then the CTHW + silanization treatments were applied.
